# Oxymetholone‐Associated Cholestatic Drug‐Induced Liver Injury With Profound Hyperbilirubinaemia: A Longitudinal Case Report

**DOI:** 10.1155/crhe/2519455

**Published:** 2026-09-11

**Authors:** Hatem Mousa Taha, Baha’ Husam Khalifa, Majd Y. Hassan, Khaled Hatem Taha

**Affiliations:** ^1^ Faculty of Medicine and Health Sciences, An-Najah National University, P. O. Box 7, Nablus, State of Palestine, upm.edu.my; ^2^ Department of Internal Medicine, Palestine Medical Complex, Ramallah, State of Palestine

**Keywords:** anabolic–androgenic steroids, cholestasis, drug-induced liver injury, hyperbilirubinaemia, oxymetholone, renal function, RUCAM

## Abstract

**Background:**

Cholestatic liver injury from 17α‐alkylated anabolic–androgenic steroids is established. We report a longitudinally documented case that emphasises phenotype definition and the limits of agent‐specific attribution.

**Case Presentation:**

A 26‐year‐old man presented on Day 29 after self‐administering oral oxymetholone 50 mg/day with intramuscular testosterone enanthate 250 mg/week. Peak total bilirubin was 24.6 mg/dL (direct bilirubin 21.3 mg/dL), alanine aminotransferase 5.6 × the upper limit of normal (ULN) and alkaline phosphatase 2.9 × ULN; the R‐ratio was 1.91, indicating cholestatic injury. International normalised ratio (≤ 1.04), albumin (≥ 4.1 g/dL) and creatinine (0.8–0.9 mg/dL) remained preserved. Ultrasonography with hepatic Doppler showed a nondilated biliary tree (common bile duct 1–2 mm), patent visualised vessels and no sonographic evidence of obstruction. The available viral, autoimmune, metabolic and haemolytic evaluation identified no alternative cause. Updated RUCAM assessment was limited by ursodeoxycholic acid use during dechallenge and incomplete molecular virology; concomitant testosterone and absent chemical verification of the products precluded definitive single‐agent attribution. Both agents were withdrawn on Day 30; ursodeoxycholic acid and antipruritic therapies were used as supportive care.

**Outcome:**

Total bilirubin decreased by 69.1% by Day 57 and by 91.5% by Day 85. Pruritus resolved, daily function returned and renal and hepatic synthetic function remained preserved.

**Conclusion:**

This report provides granular 8‐week follow‐up of an established phenotype. The course supports oxymetholone as the principal suspect, but recovery cannot be attributed to supportive pharmacotherapy in this uncontrolled case.

## 1. Introduction

Drug‐induced liver injury (DILI) is an important cause of acute liver‐test abnormalities, and anabolic–androgenic steroids (AAS), particularly 17α‐alkylated oral compounds, are a well‐recognised cause of cholestatic injury [1–3]. Oxymetholone is associated with the classic “bland cholestasis” phenotype: severe conjugated hyperbilirubinaemia and pruritus with disproportionately modest aminotransferase elevation and generally preserved hepatic synthetic function [3]. This entity is not novel. Registry and cohort studies have characterised the phenotype, its often‐protracted course, the risk of acute kidney injury and the problem of undeclared anabolic agents in bodybuilding products [4–6].

The diagnostic principles applied to suspected DILI—careful exposure history, biochemical injury‐pattern classification, imaging for structural biliary disease, systematic evaluation for competing causes and structured causality assessment—are established in international guidance and in the DILI phenotype‐standardisation literature [7–11]. Published AAS‐associated cases and series have applied these elements to varying degrees [6, 12–14]. Accordingly, this report does not propose a new diagnostic pathway.

The purpose of the present report is educational and descriptive: to provide detailed longitudinal characterisation of a severe oxymetholone‐associated cholestatic phenotype across five time points, including direct bilirubin, hepatic synthetic function and renal function; to distinguish the likely contribution of oxymetholone from concomitant testosterone enanthate and to state explicitly the limits of exposure verification and of structured causality scoring. This emphasis on transparent case‐level data is relevant because some published AAS reports provide only peak biochemical values or incomplete longitudinal renal and conjugated‐bilirubin data [4–6, 12–14].

## 2. Case Presentation

### 2.1. History and Exposure

A 26‐year‐old previously healthy male gymnasium instructor presented with a 4‐day history of progressive jaundice, dark urine, pale stools and debilitating generalised pruritus (approximately Days 25–28 from AAS initiation). There was no fever, abdominal pain, weight loss, prior hepatic illness or family history of liver disease. He reported no alcohol use.

Height, weight and body mass index at the index presentation were not documented in the available contemporaneous clinical record and were therefore not retrospectively estimated.

The exposure history was obtained in a nonjudgemental and confidentiality‐assured manner and was revisited during follow‐up, with specific attention to concomitant medications, supplements and performance‐enhancing agents. The documented performance‐enhancing exposures were oral oxymetholone 50 mg/day and intramuscular testosterone enanthate 250 mg/week, self‐administered for the preceding 29 days in an unsupervised bodybuilding context (Day 0 = AAS initiation; Day 29 = hospital presentation). No additional medication, herbal product, dietary supplement or performance‐enhancing exposure was reported during the revisited history. However, exposure ascertainment relied on patient reports, and no product packaging or residual material was available for chemical analysis; undisclosed coexposures, product mislabelling or adulteration, therefore, cannot be completely excluded. No preexposure liver‐function tests were available.

### 2.2. Physical Examination

The patient was haemodynamically stable (BP 128/78 mmHg, HR 72 bpm, temperature 36.8°C, SpO_2_ 98%). An examination revealed prominent scleral icterus, generalised jaundice and diffuse pruritic excoriations. Mild nontender hepatomegaly was present. There was no clinically appreciable splenomegaly, ascites, asterixis or other evidence of hepatic encephalopathy.

### 2.3. Investigations and Differential Diagnosis

**FIGURE 1:**
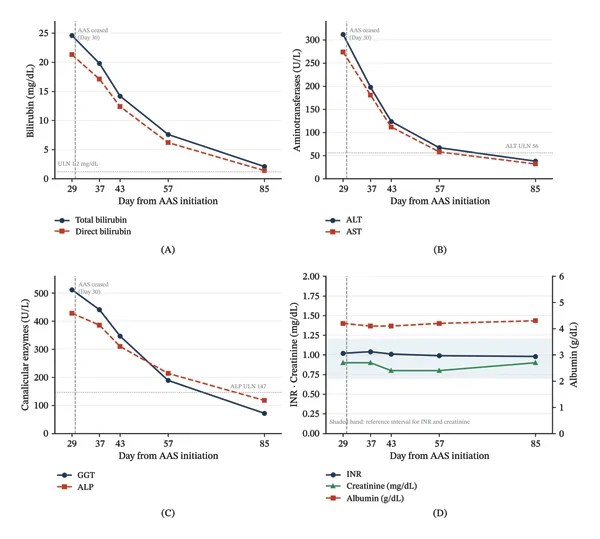
Longitudinal biochemical trajectory across the 8‐week observation window (Day 29 to Day 85 from AAS initiation; both AAS agents withdrawn on Day 30). Panel A: total and direct bilirubin. Panel B: ALT and AST. Panel C: canalicular enzymes (GGT and ALP). Panel D: preserved organ function, displaying INR, serum creatinine (left axis, with the reference interval shaded) and serum albumin (right axis) across the same time points. The figure contrasts profound cholestasis with preserved measured hepatic synthetic and renal function. AAS: anabolic–androgenic steroids; ALP: alkaline phosphatase; ALT: alanine aminotransferase; AST: aspartate aminotransferase; GGT: gamma‐glutamyl transferase; INR: international normalised ratio; ULN: upper limit of normal. (A) Bilirubin, (B) aminotransferase, (C) canalicular enzymes and (D) preserved organ function.

Presentation biochemistry (Day 29) showed a marked cholestatic injury pattern with preserved hepatic synthetic and renal function (Table 1; Figure 1). Peak total bilirubin was 24.6 mg/dL with a direct fraction of 21.3 mg/dL (86.6% conjugated), representing 20.5 × and 53.3 × ULN, respectively. GGT was 10.7 × ULN, ALP 2.9 × ULN and ALT 5.6 × ULN. INR was 1.02, albumin 4.2 g/dL and serum creatinine 0.9 mg/dL. The R‐ratio was 1.91, classifying the injury as cholestatic (*R* ≤ 2) [7, 8, 11]. Under the international DILI Expert Working Group severity index [11], the episode was classified as moderate: ALT/ALP met DILI criteria and bilirubin exceeded 2 × ULN, but INR remained below 1.5, and there was no ascites, encephalopathy or other organ failure. The descriptor ‘profound’ refers to the degree of hyperbilirubinaemia and does not imply severe‐grade DILI under this scale.

**TABLE 1:** Serial hepatic and renal biochemistry from presentation through 8‐week follow‐up.

Parameter	Day 29 (presentation)	Day 37	Day 43	Day 57	Day 85	Reference interval
ALT (U/L)	312	198	124	67	38	7–56
AST (U/L)	274	181	112	58	32	10–40
ALP (U/L)	428	386	310	214	118	44–147
GGT (U/L)	512	441	347	189	72	9–48
Total bilirubin (mg/dL)	24.6	19.8	14.2	7.6	2.1	0.2–1.2
Direct bilirubin (mg/dL)	21.3 (86.6%)	17.1	12.4	6.2	1.4	0.0–0.4
Albumin (g/dL)	4.2	4.1	4.1	4.2	4.3	3.5–5.0
INR	1.02	1.04	1.01	0.99	0.98	0.8–1.2
Creatinine (mg/dL)	0.9	0.9	0.8	0.8	0.9	0.7–1.2

Abdominal ultrasonography with hepatic Doppler demonstrated a mildly prominent liver (15–16 cm) with smooth contour and homogeneous mildly hypoechoic parenchyma, without focal hepatic lesions. The biliary tree was not dilated, and the common bile duct measured 1‐2 mm, with no definite calculus. The gallbladder was contracted at the time of the examination, limiting its assessment; no stone or wall thickening was visualised, and repeat examination in the fasting state was advised for optimal gallbladder evaluation. The visualised hepatic, portal, splenic, superior mesenteric and inferior vena caval venous segments were patent and nondilated. Mild splenomegaly (15 cm) with preserved echotexture was noted. The pancreas was unremarkable, the kidneys showed no calculi or hydronephrosis and no abdominal or pelvic mass, significant lymphadenopathy or free fluid was identified. Overall, there was no sonographic evidence of biliary obstruction.

Mild splenomegaly was not accompanied by other features suggesting chronic liver disease or portal hypertension: The portal and splenic veins were patent and nondilated; there was no ascites, hepatic contour and echotexture were preserved, and hepatic synthetic function was normal throughout. Haemolysis was excluded biochemically. The finding was, therefore, interpreted as nonspecific in the context of an acute cholestatic illness. In the absence of ductal dilatation or of any clinical feature requiring therapeutic biliary intervention, MRCP and ERCP were not pursued, and repeat imaging was not clinically required during a promptly improving course; additional imaging would have been reconsidered had the clinical or biochemical course become atypical or nonresolving [7, 8].

The documented evaluation for competing causes included viral serologies (HAV IgM, HBsAg, anti‐HBc IgM, anti‐HCV, HEV IgM, EBV VCA IgM and CMV IgM), autoimmune markers (ANA, ASMA, anti‐LKM1, AMA and quantitative IgG), metabolic investigations (serum caeruloplasmin, 24‐h urinary copper, serum iron, ferritin and transferrin saturation, alpha‐1 antitrypsin level and phenotype and TSH) and a haemolysis screen (direct antiglobulin test, haptoglobin, LDH and peripheral blood film). The available results were nonreactive, negative or within reference limits as appropriate (Supporting Table S6). These findings identified no alternative explanation in the documented evaluation; the consequences of the tests that were not available for strict application of the updated RUCAM are addressed separately in Section 2.4.

Serum creatinine was measured at each biochemical time point and remained within the reference interval throughout (0.8–0.9 mg/dL), with no creatinine‐defined acute kidney injury. This is clinically relevant because acute kidney injury and bile‐cast nephropathy have been reported in severe AAS‐associated cholestasis [4, 15–17].

Liver biopsy was not performed. The rationale and the circumstances in which histology would have been reconsidered are discussed in Section 4.6.

**FIGURE 2:**
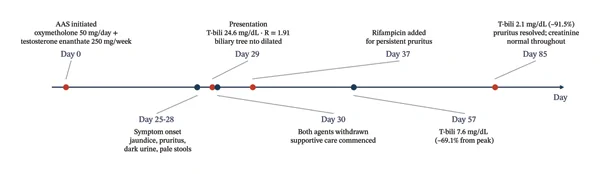
Clinical timeline of oxymetholone‐associated cholestatic DILI. Key events: AAS initiation (Day 0), symptom onset (approximately Days 25–28), presentation with peak biochemistry (Day 29), withdrawal of oxymetholone and testosterone enanthate with commencement of supportive management (Day 30), rifampicin addition for persistent pruritus (Day 37), progressive biochemical improvement (Day 57) and near‐complete recovery with preserved renal function (Day 85). All days are counted from Day 0.

Note on timing convention. Throughout this report, Day 0 denotes AAS initiation and Day 29 the day of hospital presentation (peak biochemistry). AAS were ceased on Day 30. All days in all tables are counted from Day 0; the complete clinical timeline is shown in Figure 2.

Day 0 = AAS initiation; Day 29 = hospital presentation; both AAS agents were discontinued on Day 30; Day 85 is 8 weeks after presentation and 55 days after cessation. Fold‐change normalisation is provided in Supporting Table S3 and trajectory trend analysis in Supporting Table S4 (Additional File 1). ALP: alkaline phosphatase; ALT: alanine aminotransferase; AST: aspartate aminotransferase; GGT: gamma‐glutamyl transferase; INR: international normalised ratio.

### 2.4. Causality Assessment

Causality was assessed with the 2016 updated RUCAM cholestatic/mixed subscale because the R‐ratio at the first available qualifying liver tests was 1.91 [9]. Reaudit of the submitted score identified two operational constraints. First, cholestatic dechallenge is based on ALP, not bilirubin, and treatment during dechallenge may mask the natural course; because ursodeoxycholic acid was started on the day of withdrawal, the dechallenge item was assigned 0 [9]. Second, the RUCAM‐specified molecular virology panel was incomplete.

For the prespecified primary analysis, the alternative‐cause item was assigned 0 rather than +1 or +2: HAV was excluded; the acute HBV serologic screen was negative; hepatobiliary imaging, alcohol‐related injury and recent hypotension were addressed; but HCV RNA, HEV IgG/HEV RNA and HBV DNA were not available. This yields a total of +3 (‘*possible’*) for oxymetholone (Table 2). A strict sensitivity analysis that treats fewer than five Group‐I causes as fully excluded because the listed molecular tests were not performed assigns −2 to Item 5 and yields +1 (‘*unlikely’*). The lower sensitivity score reflects missing prespecified tests and treatment‐masked dechallenge, not a documented competing diagnosis.

**TABLE 2:** Updated RUCAM (cholestatic/mixed subscale) for oxymetholone.

Item	Option applied	Documented finding	Score
1. Time to onset	5–90 days	Symptoms on Days 25–28; first available qualifying tests on Day 29	+2
2. Course of ALP	No scorable natural dechallenge	UDCA began on the day of withdrawal; ALP subsequently fell from 428 to 118 U/L	0
3. Risk factors	No risk factor	Age 26 years; no alcohol use reported; pregnancy not applicable	0
4. Concomitant drug	Compatible time to onset	Testosterone enanthate was coadministered	−1
5. Alternative causes	Five or six Group‐I causes ruled out	Acute viral serology and available competing‐cause evaluation were negative, but HBV DNA, HCV RNA, HEV IgG and HEV RNA were unavailable	0
6. Previous hepatotoxicity	Labelled reaction	Oxymetholone labelling warns of cholestatic hepatitis and jaundice [18]	+2
7. Unintentional reexposure	Not performed	Reexposure was neither performed nor recommended	0
Total		Primary analysis	+3 — possible

A separate agent‐specific worksheet was applied to testosterone enanthate (Supporting Table S5B). Its primary total was +1 (‘*unlikely’*), principally because oxymetholone is a compatible, labelled hepatotoxin and cholestasis from C‐17β‐esterified testosterone are reported only rarely [19]. Under the same strict alternative‐cause sensitivity analysis, the oxymetholone and testosterone totals were +1 and −1, respectively. These scores support greater relative plausibility for oxymetholone but do not establish absolute single‐agent causality.

The final attribution is, therefore, phrased as oxymetholone‐associated cholestatic DILI with concomitant testosterone enanthate exposure. It is supported by the compatible latency, characteristic phenotype, labelled hepatotoxicity of oxymetholone, nonobstructive imaging, available negative competing‐cause evaluation and improvement after withdrawal. Coexposure and the absence of chemical verification of either product remain material limitations.

### 2.5. Management

Management was centred on withdrawal of the suspected exposures and symptomatic care. Oxymetholone and testosterone enanthate were permanently discontinued on Day 30. UDCA 15 mg/kg/day was commenced as adjunctive therapy according to local practice. Cholestyramine 4 g twice daily was started for pruritus, and rifampicin 150 mg twice daily was added on Day 37 because pruritus remained troublesome. Rifampicin was subsequently tapered and discontinued by Day 85. These therapies are reported descriptively; their contribution to biochemical recovery cannot be determined from this uncontrolled observation. The patient was counselled regarding the hepatotoxic risks of reexposure and confirmed continued abstinence from AAS at follow‐up visits.

## 3. Outcome

All measured hepatic biochemical parameters improved after withdrawal (Table 1; Figure 1). Total bilirubin declined from 24.6 mg/dL at Day 29 to 19.8 mg/dL at Day 37, 14.2 mg/dL at Day 43, 7.6 mg/dL at Day 57 and 2.1 mg/dL at Day 85 (91.5% reduction from the Day‐29 peak). Direct bilirubin fell in parallel from 21.3 to 1.4 mg/dL. ALT, AST, ALP and GGT also declined progressively, while albumin and INR remained within their reference intervals.

Renal function remained preserved at all five reported time points, with serum creatinine 0.8–0.9 mg/dL and no creatinine‐defined acute kidney injury, and without any requirement for albumin infusion, renal replacement therapy or plasma exchange. This negative finding is clinically relevant because renal injury, including bile‐cast nephropathy, has been described in severe AAS‐associated cholestasis [4, 15–17].

Pruritus improved substantially by Day 43 and had nearly resolved by Day 57. At Day 85, the patient was asymptomatic, had returned to normal daily activities and confirmed continued abstinence from AAS. Total bilirubin was near normal (2.1 mg/dL; ULN 1.2 mg/dL), although complete biochemical normalisation beyond the 8‐week follow‐up window was not documented.

The patient’s own account at follow‐up was of complete symptomatic recovery and return to normal daily function. He reported that he had been unaware of the hepatotoxic potential of oral anabolic agents before this episode, accepted the association with his steroid use and confirmed that he had not resumed them. A formal written patient‐perspective statement was not obtained separately; the above is documented in the treating clinicians’ records.

## 4. Discussion

### 4.1. Clinical Phenotype and Diagnostic Context

The combination of profound conjugated hyperbilirubinaemia, severe pruritus, a cholestatic R‐ratio, disproportionately modest aminotransferase elevation and preserved synthetic function is highly characteristic of the AAS‐associated bland cholestasis phenotype described in the historical and contemporary literature [3–6]. The presentation can clinically resemble extrahepatic obstruction because jaundice, pale stools, dark urine and cholestatic enzymes occur in both settings. In this patient, however, ultrasonography demonstrated a nondilated biliary tree with a common bile duct of 1‐2 mm, an unremarkable pancreas and no mass lesion, and thus no sonographic evidence of biliary obstruction.

The value of this report is not the diagnostic sequence itself. The sequence—phenotype classification, imaging, investigation of competing causes, exposure history and causality assessment—is established practice [7, 8]. The useful feature here is the completeness of the longitudinal description, including direct bilirubin, INR, albumin and creatinine at five time points.

### 4.2. Applying Established Diagnostic Standards in Practice

The case illustrates how established DILI principles were applied to a specific clinical configuration. R‐ratio calculation classified the biochemical pattern as cholestatic. Ultrasonography with Doppler showed no biliary dilatation or vascular obstruction. The documented workup investigated major viral, autoimmune, metabolic and haemolytic alternatives. The exposure history was revisited because underdisclosure and product mislabelling are recognised problems in bodybuilding‐associated liver injury [5, 20, 21].

None of these elements is diagnostic in isolation. Their convergence, together with improvement after withdrawal, supported DILI as the working diagnosis. MRCP, ERCP and liver biopsy were not treated as tests that must always be avoided; rather, they were not pursued in this patient because there was no sonographic evidence of ductal obstruction, there was no therapeutic indication for ERCP and the subsequent course was favourable. Reassessment would have been appropriate had the trajectory become atypical or persistent [7, 8].

### 4.3. Oxymetholone Versus Testosterone Enanthate

The manuscript should not be read as implying that intramuscular testosterone esters carry a cholestatic risk comparable to oral 17α‐alkylated androgens. Oxymetholone is a 17α‐alkylated oral androgen with a well‐established association with cholestatic liver injury [3, 19]. By contrast, testosterone enanthate is non–17α‐alkylated and is not the classic exposure underlying this phenotype. Published AAS cholestasis cohorts are dominated by oral 17α‐alkylated or designer androgen exposures [4–6].

The observed phenotype—marked conjugated hyperbilirubinaemia with a relatively modest ALT rise—is substantially more characteristic of 17α‐alkylated AAS cholestasis than of parenteral testosterone exposure [3–6, 19, 22]. Nevertheless, testosterone enanthate was temporally coadministered, and an additive contribution cannot be excluded; it was accordingly retained as a scored concomitant exposure in the causality assessment. For this reason, the final diagnosis is phrased as oxymetholone‐associated rather than as an unequivocally proven single‐agent reaction.

### 4.4. Renal Function and Bile‐Cast Nephropathy

Acute kidney injury is a recognised complication of severe cholestatic AAS DILI. Registry data have associated greater hyperbilirubinaemia and a cholestatic phenotype with higher AKI risk, and biopsy‐proven bile‐cast nephropathy has been reported in bodybuilders using anabolic agents, occasionally requiring plasma exchange [4, 15–17]. We deliberately avoid applying a bilirubin cutoff to individual risk because cohort‐derived discriminating values are not validated as universal clinical thresholds.

The important patient‐level observation is that, despite a peak total bilirubin of 24.6 mg/dL, serum creatinine remained 0.8‐0.9 mg/dL across five measurements. Reporting this explicitly addresses a clinically relevant complication without implying that a particular bilirubin value predicts an individual outcome and supports the practical point that renal function should be documented rather than assumed in these patients.

### 4.5. Natural History and the Limited Inference Possible for Supportive Therapy

AAS‐associated cholestasis commonly improves after withdrawal, but recovery may take weeks to months and can be protracted [5, 6, 23–25]. The 91.5% bilirubin reduction by Day 85 in this case lies within that known natural history. Drug withdrawal is the primary management step.

UDCA was used as adjunctive therapy, while cholestyramine and rifampicin were used for pruritus. No specific pharmacological therapy has established efficacy in cholestatic DILI; current guidance emphasises withdrawal of the implicated agent together with symptomatic management, and the evidence for UDCA in this setting is limited [7, 8]. Evidence for other bile‐acid–directed antipruritic strategies derives largely from chronic cholestatic disorders rather than from AAS‐associated DILI and should not be interpreted as evidence of disease modification here [26]. Accordingly, the temporal association between adjunctive therapy and biochemical improvement cannot establish treatment efficacy in this case. The same applies to rifampicin: Its use was symptomatic and should not be interpreted as causing the subsequent bilirubin decline. As noted in Section 2.4, the early use of adjunctive therapy also has a methodological consequence, in that it precluded positive scoring of the dechallenge component of the updated RUCAM.

### 4.6. Why Liver Biopsy Was Deferred—and When It Would Be Reconsidered

Liver biopsy is not routinely required in every suspected DILI case. It is most useful when the diagnosis remains uncertain, when serology or clinical features suggest a competing diagnosis such as autoimmune hepatitis, or when injury progresses or fails to resolve after withdrawal [7, 8, 10]. In this patient, the characteristic phenotype, compatible exposure, nonobstructive imaging, broad negative documented evaluation and subsequent biochemical improvement made histology unlikely to alter immediate management.

This decision was conditional on the favourable trajectory. Persistent or progressive cholestasis, diagnostic uncertainty, a superimposed process or concern for bile‐duct loss would have lowered the threshold for biopsy. Ductopenia and vanishing bile duct syndrome are uncommon but important outcomes of severe cholestatic DILI; bile‐duct loss on biopsy is associated with chronicity and adverse outcome [27]. The absence of histology means that early bile‐duct injury cannot be formally excluded, although substantial biochemical improvement by Day 85 argues against an evolving severe ductopenic course.

### 4.7. Position Within the Existing Literature

The phenotype and course are consistent with an established literature rather than being exceptional in kind. Earlier case series document severe cholestasis after anabolic or designer steroids [12–14]. Registry and prospective‐cohort data have subsequently clarified the typical disproportionate hyperbilirubinaemia, protracted course, renal complications and the problem of undeclared androgen exposure [4–6]. The present case adds value primarily through the granularity of its serial data and by explicitly separating what is documented from what cannot be independently verified. Prolonged AAS exposure is also associated with other hepatobiliary complications, including hepatocellular adenoma, and rare severe AAS‐associated DILI has required liver transplantation [28, 29]. Table 3 situates the present case within this representative literature, and Table 4 distils the corresponding practical teaching points for clinical practice.

**TABLE 3:** Present case in the context of representative AAS‐associated cholestatic liver injury literature.

Source	Design/exposure	Main relevance to the present report
Stimac et al. [14]	Case series; various AAS	Early description of AAS‐associated toxic/cholestatic hepatitis
Kafrouni et al. [12]	Case series; AAS‐containing supplements	Severe cholestasis linked to bodybuilding/dietary products
Shah et al. [13]	Five methasteron cases	Clinicopathologic bland cholestatic phenotype
El Sherrif et al. [22]	AAS supplement cases	Canalicular transporter findings supporting acquired cholestatic mechanisms
Robles‐Diaz et al. [4]	Registry cohort	Distinct AAS DILI phenotype and AKI risk
Stolz et al. [5]	Prospective DILIN cohort (44 men)	Severe and protracted cholestasis; undeclared anabolic ingredients
Abeles et al. [6]	Two cases + review of 50 additional cases	Updated‐RUCAM analysis, natural history and frequent kidney injury
Present case	Oxymetholone + testosterone enanthate	Five serial measurements of direct bilirubin, synthetic function and creatinine; nondilated biliary tree on ultrasonography with Doppler (CBD 1–2 mm); item‐by‐item updated‐RUCAM audit; explicit exposure‐verification limitations

**TABLE 4:** Practical teaching points illustrated by this case.

Clinical observation	Interpretation
Total bilirubin 24.6 mg/dL; direct bilirubin 21.3 mg/dL; *R* = 1.91	Severe conjugated cholestatic phenotype with relatively modest aminotransferase elevation
INR ≤ 1.04 and albumin ≥ 4.1 g/dL throughout	Severe jaundice occurred without deterioration in measured hepatic synthetic function
CBD 1–2 mm; biliary tree not dilated; no definite calculus; unremarkable pancreas	No sonographic evidence of biliary obstruction; invasive biliary testing was not clinically indicated in the observed configuration
Creatinine 0.8‐0.9 mg/dL at five time points	Renal function remained preserved despite severe cholestasis; renal data should be reported explicitly because acute kidney injury and bile‐cast nephropathy are recognised in this literature
Oxymetholone plus testosterone enanthate	Oxymetholone is the principal suspect, but coexposure prevents absolute single‐agent attribution
Exposure history revisited; no chemical analysis of products	Patient report is informative but cannot exclude undisclosed exposure, mislabelling or adulteration
Marked improvement after withdrawal; UDCA and antipruritics used concurrently	Improvement is consistent with natural dechallenge; treatment efficacy cannot be inferred from a single case, and early adjunctive therapy also precludes positive scoring of the RUCAM dechallenge item

### 4.8. Limitations

Several limitations are important. First, this is a single case and cannot validate a diagnostic or management strategy. Second, no preexposure liver‐function tests were available. Third, baseline height, weight and BMI were not documented contemporaneously and were not retrospectively estimated. Fourth, testosterone enanthate was coadministered, so an additive contribution cannot be excluded. Fifth, exposure ascertainment relied on patient report; the products were unavailable for chemical analysis, and undisclosed coexposure, mislabelling or adulteration cannot be excluded. Sixth, the available virologic evaluation did not include every molecular test prespecified by the updated RUCAM, and UDCA was started during dechallenge; these two circumstances constrain the attainable causality score, as detailed in Section 2.4. Seventh, liver biopsy was not performed, so early bile‐duct injury cannot be formally excluded. Finally, follow‐up ended at 8 weeks with total bilirubin at 2.1 mg/dL, near normal but not fully normalised; longer follow‐up would have better documented complete resolution and excluded late chronic injury.

## 5. Conclusions

This case documents oxymetholone‐associated cholestatic DILI with profound conjugated hyperbilirubinaemia, preserved hepatic synthetic and renal function, a nondilated biliary tree and substantial biochemical improvement during 8‐week follow‐up. Oxymetholone is the principal suspect, but concomitant testosterone enanthate and absent product authentication require cautious causal language. The primary updated‐RUCAM total was +3 (possible); a strict missing‐data sensitivity analysis yielded +1 (*unlikely*), underscoring that the instrument was constrained by incomplete molecular virology and treatment during dechallenge.

The report does not propose a novel diagnostic algorithm. Its contribution is the transparent longitudinal characterisation of an established AAS‐associated phenotype, including serial direct bilirubin and creatinine, source‐verified imaging, an item‐by‐item causality audit with an explicit missing‐data sensitivity analysis, separation of documented from unverified exposures and a clear account of why invasive investigation was deferred in this patient. Decisions regarding additional imaging or liver biopsy remain individualised and should be reconsidered if the clinical course is atypical, persistent or progressive.

**Nomenclature**
AAS: Anabolic–androgenic steroids
AKI: Acute kidney injury
ALP: Alkaline phosphatase
ALT: Alanine aminotransferase
AST: Aspartate aminotransferase
CBD: Common bile duct
DILI: Drug‐induced liver injury
DILIN: Drug‐induced liver injury network
EASL: European Association for the Study of the Liver
ERCP: Endoscopic retrograde cholangiopancreatography
GGT: Gamma‐glutamyl transferase
INR: International normalised ratio
MRCP: magnetic resonance cholangiopancreatography
RUCAM: Roussel Uclaf Causality Assessment Method
UDCA: Ursodeoxycholic acid
ULN: Upper limit of normal
VBDS: Vanishing bile duct syndrome

## Author Contributions

Hatem Mousa Taha: conceptualisation, data curation, formal analysis, investigation, methodology, project administration, supervision, writing–original draft and writing–review and editing. Baha’ Husam Khalifa: data curation, investigation, resources and writing–review and editing. Majd Y. Hassan: data curation, investigation, resources and writing–review and editing. Khaled Hatem Taha: formal analysis, investigation, visualisation and writing–review and editing.

## Funding

The authors received no specific funding for this work.

## Disclosure

All authors approved the final version of the manuscript and agreed to be accountable for all aspects of the work.

## Ethics Statement

This study was conducted in accordance with the Declaration of Helsinki. The Palestine Medical Complex Research Governance Office confirmed that formal ethics committee review was not required for publication of anonymised single‐patient case reports in which written informed consent has been obtained from the patient. All identifying information was removed before analysis and reporting. Written informed consent for publication was obtained from the patient; the signed form is securely archived by the corresponding author and is available to the journal on request.

## Conflicts of Interest

The authors declare no conflicts of interest.

## Supporting Information

Additional supporting information can be found online in the Supporting Information section.

## Supporting information


**Supporting Information 1** Supporting Figure S1: Stepwise symptomatic management of cholestatic pruritus. The figure is presented as general supportive‐care context and not as evidence that antipruritic therapy modified the biochemical course. Additional File 1: Supplementary Analytical Dataset containing six supplementary table groups: S1 (complete clinical timeline), S2 (serial absolute biochemical values), S3 (fold‐change normalisation), S4 (percentage change from the presentation peak), S5A–S5B (item‐by‐item and agent‐specific updated‐RUCAM worksheets) and S6 (documented exclusionary workup).

**Supporting Information 2** CARE 2519455_CARE_Checklist.docx.

## Data Availability

All relevant data supporting the findings of this case report are included in the article and its Supporting Information.
