# Supplementary material for: Oxymetholone‐Associated Cholestatic Drug‐Induced Liver Injury With Profound Hyperbilirubinaemia: A Longitudinal Case Report
Source: Case Reports Hepatol. 2026 Sep 11;2026:2519455. doi: 10.1155/crhe/2519455 (PMC13563310; doi:10.1155/crhe/2519455)
Supplement: Supplementary file 1 [file CRHE-2026-2519455-s001.docx]

**CARE Case Report Checklist (Final Revision)**

Manuscript title: Oxymetholone-Associated Cholestatic Drug-Induced Liver Injury with Profound Hyperbilirubinaemia: A Longitudinal Case Report
Manuscript ID: 2519455
Journal: Case Reports in Hepatology
Authors: Hatem Mousa Taha; Baha’ Husam Khalifa; Majd Y. Hassan; Khaled Hatem Taha

| **Item** | **CARE criterion** | **Location / final status** |
| --- | --- | --- |
| **1** | Title identifies the report as a case report | Title includes ‘A Longitudinal Case Report’ |
| **2** | Keywords identify diagnoses/interventions | Keywords: DILI; oxymetholone; AAS; cholestasis; hyperbilirubinaemia; RUCAM; renal function |
| **3a–d** | Abstract: background, case, diagnosis/intervention/outcome, lesson | Structured abstract; self-contained; citation-free; fewer than 300 words |
| **4** | Introduction explains value in existing literature | Section 1; explicit statement that phenotype and diagnostic principles are established |
| **5a–d** | Patient information and relevant history | Section 2.1; demographics, symptoms, medical/family/alcohol/exposure history; unavailable BMI and pre-exposure tests stated |
| **6** | Clinical findings and important investigations | Sections 2.2–2.3; Tables 1–2; Figure 1; Supplementary Tables S2, S3 and S6 |
| **7** | Timeline | Figure 2 and Supplementary Table S1; Day 0 convention; Day 85 = eight weeks after presentation and 55 days after cessation |
| **8a–d** | Diagnostic assessment, differential, challenges and prognosis | Sections 2.3–2.4 and 4.1–4.3; RUCAM primary and strict sensitivity analyses; Supplementary Tables S5A–S5B and S6 |
| **9** | Therapeutic interventions | Section 2.5: permanent withdrawal; adjunctive UDCA; cholestyramine and rifampicin for pruritus; counselling |
| **10a–c** | Follow-up, outcomes, complications and patient-reported outcomes | Section 3; Table 1; Figure 1; preserved renal function; symptom resolution; patient account |
| **11** | Discussion of strengths, limitations and literature | Sections 4.1–4.8; eight explicit limitations; conditional biopsy rationale |
| **12** | Patient perspective and informed consent | Abstract/Section 3; written consent for publication archived and available to the journal on request |
| **13** | Funding | Funding section: no specific funding |
| **14** | Conflicts of interest | Conflicts of Interest section: none declared |

# Revision-specific notes

- Causality: the original +7 updated-RUCAM total was withdrawn. The final primary total for oxymetholone is +3 (possible); strict missing-data sensitivity is +1 (unlikely). A separate testosterone-enanthate worksheet yields lower totals.
- Severity: International DILI Expert Working Group grade corrected to moderate; the title uses ‘profound hyperbilirubinaemia’ rather than severe-grade DILI.
- Imaging: common bile duct corrected against the source report to 1–2 mm; the manuscript states ‘no sonographic evidence of biliary obstruction’.
- Timing: Day 85 is eight weeks after presentation and 55 days after withdrawal.
- Figures: Figure 1, Figure 2 and the graphical abstract are original author-generated figures based on the patient data; no third-party material is reproduced.
