# Supplementary material for: Oxymetholone‐Associated Cholestatic Drug‐Induced Liver Injury With Profound Hyperbilirubinaemia: A Longitudinal Case Report
Source: Case Reports Hepatol. 2026 Sep 11;2026:2519455. doi: 10.1155/crhe/2519455 (PMC13563310; doi:10.1155/crhe/2519455)
Supplement: Supplementary file 2 [file CRHE-2026-2519455-s002.docx]

**ADDITIONAL FILE 1 — Supplementary Analytical Dataset**

Oxymetholone-Associated Cholestatic Drug-Induced Liver Injury with Profound Hyperbilirubinaemia: A Longitudinal Case Report

Hatem Mousa Taha; Baha’ Husam Khalifa; Majd Y. Hassan; Khaled Hatem Taha

Timing convention: Day 0 = AAS initiation; Day 29 = hospital presentation (peak biochemistry); Day 30 = cessation of oxymetholone and testosterone enanthate. All days are counted from Day 0.

# Contents

•	Supplementary Table S1. Complete clinical and analytical timeline

•	Supplementary Table S2. Serial absolute biochemical values

•	Supplementary Table S3. Fold-change normalisation (×ULN)

•	Supplementary Table S4. Percentage change from the presentation peak

•	Supplementary Tables S5A–S5B. Updated-RUCAM worksheets and agent-specific comparison

•	Supplementary Table S6. Documented exclusionary workup

**Supplementary Table S1. Complete Clinical and Analytical Timeline**

| **Day** | **Event** | **Details** |
| --- | --- | --- |
| **Day 0** | AAS initiated | Oral oxymetholone 50 mg/day plus intramuscular testosterone enanthate 250 mg/week; unsupervised bodybuilding context; no pre-exposure liver tests available. |
| **Days 25–28** | Symptom onset | Progressive jaundice, dark urine, pale stools and debilitating generalised pruritus. |
| **Day 29** | Hospital presentation | Clinical assessment, biochemical investigations, abdominal ultrasonography with hepatic Doppler and competing-cause evaluation. Exposure history obtained and subsequently revisited. |
| **Day 30** | Both AAS agents withdrawn; supportive treatment commenced | Oxymetholone and testosterone enanthate discontinued. UDCA 15 mg/kg/day and cholestyramine 4 g twice daily commenced. |
| **Day 37** | Rifampicin added | Rifampicin 150 mg twice daily added for persistent pruritus; biochemical reassessment performed. |
| **Day 43** | Reassessment | Total bilirubin 14.2 mg/dL; pruritus substantially improved. |
| **Day 57** | Further biochemical improvement | Total bilirubin 7.6 mg/dL (69.1% below the Day-29 peak); rifampicin taper commenced. |
| **Day 85** | Near-complete biochemical recovery | Total bilirubin 2.1 mg/dL (91.5% below peak); pruritus resolved; rifampicin discontinued; patient asymptomatic and confirmed continued abstinence from AAS. |

AAS: anabolic–androgenic steroids; UDCA: ursodeoxycholic acid.

**Supplementary Table S2. Serial Absolute Biochemical Values**

| **Parameter** | **Day 29** | **Day 37** | **Day 43** | **Day 57** | **Day 85** | **Reference interval** |
| --- | --- | --- | --- | --- | --- | --- |
| ALT (U/L) | 312 | 198 | 124 | 67 | 38 | 7–56 |
| AST (U/L) | 274 | 181 | 112 | 58 | 32 | 10–40 |
| ALP (U/L) | 428 | 386 | 310 | 214 | 118 | 44–147 |
| GGT (U/L) | 512 | 441 | 347 | 189 | 72 | 9–48 |
| Total bilirubin (mg/dL) | 24.6 | 19.8 | 14.2 | 7.6 | 2.1 | 0.2–1.2 |
| Direct bilirubin (mg/dL) | 21.3 | 17.1 | 12.4 | 6.2 | 1.4 | 0.0–0.4 |
| Albumin (g/dL) | 4.2 | 4.1 | 4.1 | 4.2 | 4.3 | 3.5–5.0 |
| INR | 1.02 | 1.04 | 1.01 | 0.99 | 0.98 | 0.8–1.2 |
| Creatinine (mg/dL) | 0.9 | 0.9 | 0.8 | 0.8 | 0.9 | 0.7–1.2 |
| Haptoglobin (g/L) | 1.1 | 1.2 | 1.3 | 1.4 | 1.5 | 0.3–2.0 |

Day 85 corresponds to eight weeks after presentation and 55 days after cessation of both agents on Day 30. The AST/ALT ratio at presentation was 0.88.

**Supplementary Table S3. Fold-Change Normalisation (×ULN)**

| **Parameter** | **Day 29** | **Day 37** | **Day 43** | **Day 57** | **Day 85** | **ULN** |
| --- | --- | --- | --- | --- | --- | --- |
| ALT | 5.57× | 3.54× | 2.21× | 1.20× | 0.68× | 56 U/L |
| AST | 6.85× | 4.53× | 2.80× | 1.45× | 0.80× | 40 U/L |
| ALP | 2.91× | 2.63× | 2.11× | 1.46× | 0.80× | 147 U/L |
| GGT | 10.67× | 9.19× | 7.23× | 3.94× | 1.50× | 48 U/L |
| Total bilirubin | 20.50× | 16.50× | 11.83× | 6.33× | 1.75× | 1.2 mg/dL |
| Direct bilirubin | 53.25× | 42.75× | 31.00× | 15.50× | 3.50× | 0.4 mg/dL |

R-ratio at presentation = (ALT ÷ ULN) ÷ (ALP ÷ ULN) = 5.57 ÷ 2.91 = 1.91, defining a cholestatic pattern (R ≤ 2) and determining the use of the cholestatic/mixed subscale of the updated RUCAM (Table S5A). ULN: upper limit of normal.

**Supplementary Table S4. Percentage Change from the Day-29 Peak**

| **Parameter** | **Day 37** | **Day 43** | **Day 57** | **Day 85** |
| --- | --- | --- | --- | --- |
| ALT | −36.5% | −60.3% | −78.5% | −87.8% |
| AST | −33.9% | −59.1% | −78.8% | −88.3% |
| ALP | −9.8% | −27.6% | −50.0% | −72.4% |
| GGT | −13.9% | −32.2% | −63.1% | −85.9% |
| Total bilirubin | −19.5% | −42.3% | −69.1% | −91.5% |
| Direct bilirubin | −19.7% | −41.8% | −70.9% | −93.4% |

These percentages describe the observed biochemical trajectory only. They are not used to assign updated-RUCAM dechallenge points, because ursodeoxycholic acid was initiated on the day of withdrawal and the instrument specifies that treatment during the dechallenge phase permits only a score of 0 for that item (Table S5A).

**Supplementary Table S5A. Updated-RUCAM Worksheet for Oxymetholone (Cholestatic/Mixed Subscale)**

The cholestatic/mixed subscale was used because the R-ratio at the first available qualifying liver tests was 1.91. The primary score deliberately awards no positive points for the alternative-cause item because the RUCAM-specified molecular virology panel was incomplete.

| **#** | **Item and applied option** | **Score** | **Patient data and rationale** |
| --- | --- | --- | --- |
| **1** | Time to onset: 5–90 days | +2 | Symptoms Days 25–28; first available qualifying tests Day 29 |
| **2** | Course of ALP: no scorable natural dechallenge | 0 | UDCA commenced on the day of withdrawal; treatment during dechallenge may mask the natural course. ALP nevertheless fell from 428 to 118 U/L by Day 85 |
| **3** | Risk factors: none | 0 | Age 26 years; no alcohol use reported; pregnancy not applicable |
| **4** | Concomitant drug with compatible time to onset | −1 | Testosterone enanthate was co-administered |
| **5** | Five or six group-I causes ruled out | 0 | HAV excluded; acute HBV serology negative; imaging, alcohol and hypotension addressed. HBV DNA, HCV RNA, HEV IgG and HEV RNA were unavailable; therefore +1 or +2 is not claimed |
| **6** | Previous hepatotoxicity: labelled reaction | +2 | Oxymetholone labelling warns of cholestatic hepatitis and jaundice |
| **7** | Unintentional re-exposure: not performed | 0 | Re-exposure was not performed or recommended |
|  | Primary total | +3 | Possible |

Strict sensitivity analysis. If item 5 is assigned −2 because fewer than five group-I causes are regarded as fully excluded without every molecular test listed in the updated RUCAM, the total is +1 ("unlikely"). The difference is a missing-data sensitivity analysis, not evidence that a competing diagnosis was identified.

**Supplementary Table S5B. Agent-Specific Updated-RUCAM Comparison**

| **Item** | **Oxymetholone** | **Testosterone enanthate** | **Rationale for difference** |
| --- | --- | --- | --- |
| **Time to onset** | +2 | +2 | Both agents had a compatible latency |
| **ALP dechallenge** | 0 | 0 | UDCA commenced at withdrawal of both agents |
| **Risk factors** | 0 | 0 | No RUCAM risk factor |
| **Concomitant exposure** | −1 | −2 | Testosterone is a compatible co-exposure for oxymetholone; oxymetholone is a compatible labelled hepatotoxin for testosterone |
| **Alternative causes** | 0 | 0 | Same incomplete RUCAM-specified molecular virology panel |
| **Previous hepatotoxicity** | +2 | +1 | Labelled cholestatic injury for oxymetholone; cholestasis only rarely reported with C-17β-esterified testosterone |
| **Re-exposure** | 0 | 0 | Not performed |
| **Primary total** | +3 (possible) | +1 (unlikely) | Relative causality favours oxymetholone |
| **Strict sensitivity total** | +1 (unlikely) | −1 (excluded) | Item 5 assigned −2 for both agents |

Interpretation. RUCAM is used here as a transparent structured aid rather than as proof of causation. Missing molecular virology, treatment during dechallenge, concomitant exposure and absent product authentication materially limit the attainable scores.

**Supplementary Table S6. Documented Exclusionary Workup**

| **Category** | **Tests / assessment documented** | **Result / interpretation** |
| --- | --- | --- |
| **Hepatitis A** | HAV IgM | Non-reactive |
| **Hepatitis B** | HBsAg; anti-HBc IgM | Non-reactive; HBV DNA not documented |
| **Hepatitis C** | Anti-HCV | Non-reactive; HCV RNA not documented |
| **Hepatitis E** | HEV IgM | Non-reactive; HEV IgG and HEV RNA not documented |
| **EBV / CMV** | EBV VCA IgM; CMV IgM | Non-reactive; paired titres and PCR not documented |
| **Autoimmune hepatitis** | ANA; ASMA; anti-LKM1; quantitative IgG | Negative / within reference limits |
| **Primary biliary cholangitis** | AMA | Negative |
| **Wilson disease** | Serum caeruloplasmin; 24-hour urinary copper | Within reference limits |
| **Iron overload** | Serum iron; ferritin; transferrin saturation | Within reference limits |
| **Alpha-1 antitrypsin deficiency** | Serum level; phenotype | Normal level; MM phenotype |
| **Thyroid dysfunction** | TSH | Within reference limits |
| **Haemolysis** | DAT; haptoglobin; LDH; peripheral blood film | No evidence of haemolysis; no schistocytes reported |
| **Renal involvement** | Serial serum creatinine at all five time points | 0.8–0.9 mg/dL; no creatinine-defined acute kidney injury |
| **Alcohol** | Clinical history; AST/ALT ratio | Patient reported no alcohol use; AST/ALT 0.88 |
| **Haemodynamic cause** | Clinical history and observations at presentation | No recent hypotensive episode; BP 128/78 mmHg at presentation |
| **Structural, biliary and vascular disease** | Abdominal ultrasonography with hepatic Doppler | Liver 15–16 cm, smooth contour, homogeneous mildly hypoechoic parenchyma; no focal lesion; biliary tree not dilated; CBD 1–2 mm; no definite calculus; gallbladder contracted at the time of examination with repeat fasting examination advised; visualised IVC, hepatic, portal, splenic and superior mesenteric venous segments patent and non-dilated; mild splenomegaly (15 cm); pancreas unremarkable; kidneys without calculi or hydronephrosis; no mass, significant lymphadenopathy or free fluid. No sonographic evidence of biliary obstruction |
| **Other medication, supplement or performance-enhancing exposure** | Exposure history revisited during follow-up with specific attention to medications, supplements and performance-enhancing agents | No additional exposure reported. No chemical analysis of the administered products was available, so undisclosed exposure, mislabelling or adulteration cannot be completely excluded |

Abbreviations. AAS: anabolic–androgenic steroids; ALP: alkaline phosphatase; ALT: alanine aminotransferase; AMA: anti-mitochondrial antibody; ANA: antinuclear antibody; ASMA: anti-smooth muscle antibody; AST: aspartate aminotransferase; CBD: common bile duct; CMV: cytomegalovirus; DAT: direct antiglobulin test; EBV: Epstein–Barr virus; GGT: gamma-glutamyl transferase; HAV: hepatitis A virus; HBsAg: hepatitis B surface antigen; HBV: hepatitis B virus; HCV: hepatitis C virus; HEV: hepatitis E virus; INR: international normalised ratio; IVC: inferior vena cava; LDH: lactate dehydrogenase; RUCAM: Roussel Uclaf Causality Assessment Method; TSH: thyroid-stimulating hormone; UDCA: ursodeoxycholic acid; ULN: upper limit of normal.
